# Association between Uric Acid and In-Hospital Heart Failure in Patients with Acute Myocardial Infarction Undergoing Percutaneous Coronary Intervention

**DOI:** 10.1155/2021/7883723

**Published:** 2021-07-08

**Authors:** Cun-Fei Liu, Kai-You Song, Wei-Ning Zhou, Yan-Jin Wei

**Affiliations:** ^1^Department of Cardiology, Linyi People's Hospital, Linyi City, Shandong Province, 276000, China; ^2^Department of Pathology, Linyi People's Hospital, Linyi City, Shandong Province, 276000, China

## Abstract

**Objective:**

To investigate the association of serum uric acid levels with in-hospital heart failure (HF) in patients with acute myocardial infarction (AMI) who are undergoing percutaneous coronary intervention (PCI).

**Methods:**

Two hundred sixteen patients with AMI who were treated with PCI were enrolled in our study. Univariate and multivariate logistic regression analyses were performed to estimate the associations between uric acid levels and the risk of in-hospital HF in AMI patients. Analyses of the areas under the receiver operating characteristic (ROC) curve were performed to determine the accuracy of uric acid levels in predicting in-hospital HF.

**Results:**

A dose-response relationship was found for the incidence of in-hospital HF and levels of uric acid, showing increased HF from the lowest to the highest tertile of uric acid. Compared with subjects in the bottom tertile, the adjusted odds ratio for in-hospital HF was 1.92 (95% CI 0.70–5.24) and 3.33 (95% CI 1.18-9.46) in the second tertile group and the third tertile group, respectively. Every 1 mg/dl increase in the serum uric acid level was associated with a 1.60-fold increased risk of incident in-hospital HF (OR, 1.60; 95% CI 1.22–2.11; *P* = 0.001). ROC curve analysis showed that the optimal cut-off value of uric acid to predict in-hospital HF was 5.75 mg/dl with a sensitivity of 69.2% and specificity of 56.3%.

**Conclusions:**

Our study showed that the serum uric acid level on admission is an independent predictor of in-hospital heart failure in patients with AMI.

## 1. Introduction

Although the treatment of acute myocardial infarction (AMI) has improved substantially during the past decade, AMI remains one of the most significant causes of annual deaths worldwide and was regarded as the largest health burden worldwide in 2030 [1]. Heart failure (HF), either early in-hospital or late postdischarge HF after AMI, is a serious complication of AMI [2, 3], and HF complicating AMI has been associated with a much higher mortality and worse prognosis than HF without AMI [4–6]. Therefore, early identification of patients with AMI who are at high risk of developing HF is necessary to reduce AMI-related major adverse cardiovascular events.

Serum uric acid is the metabolic end product of purine metabolism and is regarded as a biomarker of inflammatory response [7]. Elevated uric acid is a well-established risk factor for the development of future HF in community populations [8, 9]. However, whether elevated uric acid is associated with HF in patients hospitalized with an incident AMI remains unclear. HF is a common complication of the first AMI during hospitalization, and post-AMI HF significantly increases early mortality in AMI patients [3, 6]. Therefore, we investigated the association of serum uric acid levels with in-hospital HF in patients with AMI treated with percutaneous coronary intervention (PCI).

## 2. Methods

### 2.1. Study Populations

Our present study prospectively included 216 AMI patients who underwent PCI immediately or during the first 72 hours after admission between May 2016 and June 2017. The test power of our present sample size is 0.9994 according to computer power simulation. All patients were first diagnosed with AMI. The diagnosis of AMI was defined as follows [10]: detection of a rise in cardiac troponin and with at least one symptom out of ischaemia, significant new changes or presumed new changes in ST-segment–T wave (ST–T) or new left bundle branch block (LBBB), development of pathological Q waves on ECG, imaging evidence of new loss of viable myocardium or new regional wall motion abnormalities, or identification of an intracoronary thrombus by angiography.

Patients with a known history of heart failure or old myocardial infarction, gout, active infections, systemic inflammatory disease, or malignancy or those who did not receive intervention procedures during the first 72 hours were excluded. Of these 216 patients, 133 were diagnosed with ST-segment elevation myocardial infarction (STEMI) and 83 with non-ST-segment elevation myocardial infarction (NSTEMI). Only culprit vessel in STEMI subjects was treated, and the TIMI flow grade of all included STEMI patients was restored to TIMI flow 3 after PCI procedures. Written informed consent was obtained from all participants, and the study was approved by the Ethics Committee of Linyi People's Hospital.

The primary end point was the presentation of new-onset HF during hospitalization according to the Killip classification [11] (class I: no heart failure; class II: emergence with S3 and/or basal lung rales less than 50% of the lung fields; class III: acute pulmonary oedema; and class IV: cardiogenic shock. Killip ≥ II was considered as presentation of HF).

### 2.2. Laboratory Measurements and Echocardiography

Venous blood samples were drawn on admission for the laboratory analysis, and serum uric acid concentrations and other routine biochemical parameters were measured according to standard methods by the laboratory institutions. Transthoracic Doppler echocardiography was performed in all patients in the first 24 hours after admission to the intensive cardiac care unit. The measurements were performed using a commercially available machine (Vivid 7, GE Healthcare, USA). During hospitalization, patient history of hypertension, diabetes, dyslipidaemia, and smoking was also collected.

### 2.3. Statistical Analysis

Continuous variables are summarized as the mean with standard deviation (SD) or median (25th and 75th percentiles) according to whether the parameters were normally distributed. Continuous variables were compared using Student's *t*-test, one-way ANOVA, or nonparametric tests (Mann-Whitney or Kruskal-Wallis test) between two groups or multiple groups. Categorical variables are summarized as numbers and percentages and were compared with the chi-squared test. Univariate and multiple logistic regressions were performed to evaluate the association between uric acid and the risk of in-hospital HF post-AMI in hospitalization. The receiver operating characteristic (ROC) curve was used to determine the sensitivity and specificity of uric acid and the optimal cut-off value for predicting in-hospital HF post-AMI. A *P* value < 0.05 was considered statistically significant, and all statistical analyses were performed with SPSS software 18.0 (SPSS Inc., Chicago, IL, USA).

## 3. Results

### 3.1. Characteristics of Patients between the HF and Non-HF Groups

Two hundred and sixteen patients were included in our present study. The main features of the enrolled patients are shown in Table 1. Sixty-five patients were diagnosed with HF during hospitalization, and the prevalence of HF was 30.1% among all AMI patients. In general, patients with AMI complicated with HF were older (mean age: 70.45 vs. 61.74, *P* < 0.001), with a higher proportion of females (33.8% vs. 15.9, *P* = 0.003), and a higher prevalence of DM (47.7% vs. 26.5%, *P* = 0.002), hypertension (76.9% vs. 57.6%, *P* = 0.007), and in-hospital mortality (12.3% vs. 1.3%, *P* < 0.001). However, there was no significant difference in smoking or type of AMI (*P* = 0.057 and 0.247, respectively). Regarding the baseline laboratory parameters, the in-hospital HF group had significantly higher levels of CRP, uric acid, and BNP on admission (all *P* < 0.001) and lower TG and eGFR (*P* = 0.019 and *P* < 0.001, respectively), while there was no significant difference in white blood cell count (WBC), TC, LDL-c, HDL-c, or ALT between the HF and non-HF groups. In addition, the left ventricular ejection fraction (LVEF) in the in-hospital HF group was significantly lower than that in the non-HF group (47.72% vs. 59.08%, *P* < 0.001).

### 3.2. Characteristics of Patients according to Uric Acid Tertiles

We further evaluated the characteristics of patients according to uric acid tertiles (Table 2). The incidence rates of HF were 17.8%, 32.4%, and 40.3% in the tertile groups, respectively, and significant linear trend associations were observed across increasing tertiles (*P* = 0.011). The eGFR, TG, and TC levels were also significantly different between the groups. However, there was no substantial difference in the presence of DM, hypertension, smoking, type of AMI, or in-hospital mortality between the groups. Moreover, there was no significant difference in age, BMI, CRP, WBC counts, ALT levels, LDL-c levels, HDL-c levels, or LVEF among the three groups.

### 3.3. Association between UA and the Risk of In-Hospital HF Post-AMI

Univariate and multivariate logistic regression analyses were performed to evaluate the association of uric acid levels and the risk of in-hospital HF in AMI patients. The main outcome is shown in Table 3. Compared with subjects in the bottom tertile, the unadjusted OR of the HF group increased from 2.21 (95% CI 1.02–4.82) for the second tertile group to 3.11 (95% CI 1.45–6.67) for the third tertile group (*P* for trend = 0.004). In the multivariate analysis, the OR of in-hospital HF was still significant after adjusting for several covariates (age, sex, CRP, current smoking, HBP, DM, and eGFR): the OR of HF incidence was 1.92 (95% CI 0.70–5.24) and 3.33 (95% CI 1.18-9.46) in the second tertile group and the third tertile group, respectively. When serum uric acid was used as a continuous variable, every 1 mg/dl increase in the serum uric acid level was associated with a 1.60-fold increased risk of HF (OR, 1.60; 95% CI 1.22–2.11; *P* = 0.001). Other possible risk factors related to in-hospital HF are presented in Supplementary Materials (available here).

### 3.4. Accuracy of UA in Predicting In-Hospital HF Post-AMI

**Figure 1:**
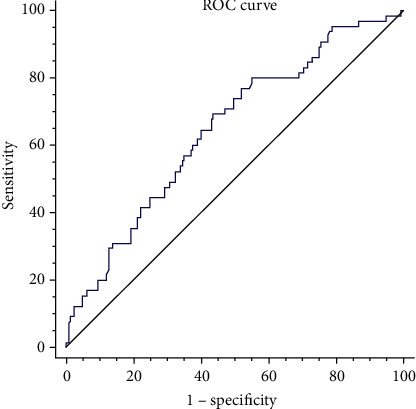
Receiver operating characteristic (ROC) curve analysis of uric acid and in-hospital HF post-AMI.

Receiver operating characteristic (ROC) curve analysis of uric acid is shown in Figure 1. The area under the curve was 0.648 (95% CI 0.569-0.727; *P* = 0.001). Although the area under the curve was relatively low, it was still considered relevant. According to the ROC curve analysis, the optimal cut-off value of uric acid to predict in-hospital HF was 5.75 mg/dl with a sensitivity of 69.2% and specificity of 56.3%.

### 3.5. The Association of In-Hospital HF and Risk of Mortality during Hospitalization

A previous study demonstrated that in-hospital HF significantly increased the rate of early mortality in patients with an incident AMI [6]. We therefore analyzed the relationship of post-AMI HF with mortality during hospitalization and found that post-AMI HF significantly increased in-hospital mortality after adjusting for age and sex (OR, 5.96; 95% CI 1.15-30.80; *P* = 0.033).

## 4. Discussion

Studies have demonstrated that heart failure (HF) commonly develops after acute myocardial infarction (AMI), and patients with AMI who develop HF during hospitalization have shown worse short-term and long-term outcomes than those without HF [4, 6]. In addition, epidemiological studies have shown a positive association between hyperuricaemia and future incident HF in community populations. One recent meta-analysis reported that the risk of incident HF in hyperuricaemia is 1.65 times higher than that in normal uric acid populations [12]. However, there is still no study regarding the association between uric acid and in-hospital HF post-AMI. Our present study demonstrated that elevated uric acid on admission significantly increased the risk of in-hospital HF in AMI patients undergoing PCI. Compared with the first tertile, the HF incidence was significantly higher in the third tertile uric acid group (40.3% vs. 17.8%). The OR of incident HF in the top uric acid tertile is 2.96 times (95% CI 1.02–8.58) higher than that in the first tertile, even after adjusting for several confounders. We also found that in-hospital HF was associated with increased mortality during hospitalization in patients with an incident AMI (OR, 5.96; 95% CI 1.15-30.80; *P* = 0.033).

To date, the underlying pathophysiological mechanism between uric acid and HF remains poorly elucidated. Uric acid is the final oxidation metabolic product of purine metabolism in the blood. Elevated serum uric acid can occur due to impaired renal elimination or excessive production by increased xanthine oxidase activity in hypoxic or ischaemic conditions [13, 14]. Ekundayo et al. [9] believed that the association between hyperuricaemia and HF is due to increased xanthine oxidase activity but not due to impaired renal excretion, as they found no association between hyperuricaemia and incident HF in CKD patients. Our present study also found a positive association between uric acid and in-hospital HF post-AMI even after adjusting for GFR. Inhibition of xanthine oxidase significantly improved left ventricular dysfunction in an animal model of HF [15]. Elevated uric acid can inhibit the generation and activity of nitric oxide, which can lead to reducing endothelium-dependent vasorelaxation and subsequent endothelial dysfunction [16]. Moreover, elevated uric acid can induce vascular smooth muscle cell proliferation and angiotensin II production via activating the renin-angiotensin system [17]. In addition, the inflammatory response may be another possible explanation for the association between uric acid and HF incidence [18]. Accordingly, Akpek et al. [19] reported that serum uric acid was correlated with hs-CRP levels and that uric acid on admission was associated with impaired coronary flow following primary PCI among patients with STEMI.

Our present study had several limitations. First, the number of subjects was not large enough, and most of the patients were male. Second, our study enrolled patients with STEMI and NSTEMI, and we did not conduct separate analyses for each AMI type because of the small sample size. Third, some potential factors, such as coronary anatomy, blushing, and microembolization, were not further analyzed due to incomplete information. Lastly, we adjusted for several potential confounding factors during our evaluations of the associations of uric acid with HF in multiple logistic regression analysis; however, residual confounding factors may still exist.

## 5. Conclusions

In conclusion, our present study suggests that uric acid is positively associated with in-hospital HF in patients with AMI. As in-hospital HF post-AMI can significantly increase short-term and long-term MACEs, patients with AMI with high uric acid levels should be treated carefully for cardiovascular events during the in-hospital and follow-up periods.

## Figures and Tables

**Table 1:** Characteristics of the study populations.

	All patients	HF (*n* = 65)	Non-HF (*n* = 151)	*P* value
Age (year)	64.36 ± 12.30	70.45 ± 11.02	61.74 ± 11.92	<0.001
Female (%)	46	22 (33.8%)	24 (15.9%)	0.003
BMI (kg/m^2^)	24.58 ± 3.68	24.75 ± 3.29	24.51 ± 3.84	0.716
Diabetes mellitus (%)	71	31 (47.7%)	40 (26.5%)	0.002
Hypertension (%)	137	50 (76.9%)	87 (57.6%)	0.007
Smoking (%)	111	24 (36.9%)	77 (51.0%)	0.057
STEMI (%)	133	44 (67.7%)	89 (58.9%)	0.247
Death during hospitalization (%)	10	8 (12.3%)	2 (1.3%)	<0.001
GRACE score	148.26 + 33.57	165.65 ± 35.80	142.20 ± 30.79	0.008
Hospitalization day	9.12 ± 4.16	12.10 ± 5.39	7.86 ± 2.67	<0.001
LVEF (%)	55.74 ± 9.58	47.72 ± 11.51	59.08 ± 6.11	<0.001
CRP (mg/l)	5.15 (1.9-16.55)	15.8 (3.4-41.5)	4.5 (1.6-10.8)	<0.001
BNP (pg/ml)	176 (80-501.5)	900 (611.5-1208.5)	113 (49.5-188.5)	<0.001
WBC (10^9^/l)	9.69 ± 3.29	9.82 ± 3.73	9.63 ± 3.09	0.699
ALT (IU/l)	39 (29-57)	43 (33.5-61.5)	37 (29-56)	0.076
UA (mg/dl)	5.87 ± 1.68	6.50 ± 1.77	5.60 ± 1.57	<0.001
eGFR (ml/min/1.73 m^2^)	83.63 ± 28.64	67.84 ± 31.87	90.43 ± 24.24	<0.001
TG (mmol/l)	1.67 ± 1.12	1.39 ± 0.59	1.79 ± 1.26	0.019
TC (mmol/l)	4.48 ± 1.06	4.33 ± 1.15	4.54 ± 1.02	0.198
LDL-c (mmol/l)	3.03 ± 0.99	2.93 ± 1.20	3.07 ± 0.89	0.370
HDL-c (mmol/l)	1.07 ± 0.31	1.07 ± 0.26	1.08 ± 0.33	0.901

Abbreviation: BMI: body mass index; STEMI: ST-segment elevation myocardial infarction; LVEF: left ventricular ejection fraction; WBC: white blood cell; ALT: alanine aminotransferase; TG: triglyceride; TC: total cholesterol; LDL-c: low-density lipoprotein cholesterol; HDL-c: high-density lipoprotein cholesterol.

**Table 2:** Characteristics of the study populations according to UA tertiles.

	Q1 (*n* = 73)	Q2 (*n* = 71)	Q3 (*n* = 72)	*P* value
Uric acid tertiles (mg/dl)	≤5.13	5.13-6.40	>6.40	
Age (year)	64.93 ± 11.59	63.45 ± 12.29	64.68 ± 13.11	0.745
Female (%)	22 (30.1%)	12 (16.9%)	12 (16.7%)	0.076
BMI (kg/m^2^)	23.69 ± 4.73	24.79 ± 3.11	25.15 ± 2.97	0.129
Diabetes mellitus (%)	27 (37.0%)	27 (38.0%)	17 (23.6%)	0.122
Hypertension (%)	44 (60.3%)	45 (63.4%)	48 (66.7%)	0.727
Smoking (%)	32 (43.8%)	37 (52.1%)	32 (44.4%)	0.543
STEMI (%)	42 (57.5%)	45 (63.4%)	46 (63.9%)	0.644
Death during hospitalization (%)	3 (4.1%)	4 (5.6%)	3 (4.2%)	0.886
Hospitalization day	8.41 ± 3.59	9.83 ± 4.70	9.16 ± 4.11	0.134
HF (Killip ≥ 2)	13 (17.8%)	23 (32.4%)	29 (40.3%)	0.011
LVEF (%)	56.96 ± 9.50	55.72 ± 9.47	54.45 ± 9.75	0.326
CRP (mg/l)	4.5 (1.9-11.2)	6.16 (1.9-24.9)	5.3 (1.9-16.25)	0.460
BNP (pg/ml)	147 (101.25-273)	176 (70-518.25)	193 (75-737.5)	0.234
WBC (10^9^/l)	9.41 ± 3.30	10.08 ± 3.35	9.59 ± 3.24	0.461
ALT (IU/l)	36 (26-55)	44 (29-58)	41.5 (31-61)	0.077
eGFR (ml/min/1.73 m^2^)	95.72 ± 28.98	83.01 ± 25.66	71.99 ± 26.37	<0.001
TG (mmol/l)	1.28 ± 0.51	1.88 ± 1.19	1.85 ± 1.38	0.001
TC (mmol/l)	4.32 ± 0.99	4.77 ± 1.15	4.34 ± 0.98	0.019
LDL-c (mmol/l)	2.95 ± 0.88	3.21 ± 1.16	2.93 ± 0.91	0.185
HDL-c (mmol/l)	1.13 ± 0.31	1.08 ± 0.34	1.01 ± 0.31	0.062

Abbreviation: BMI: body mass index; STEMI: ST-segment elevation myocardial infarction; LVEF: left ventricular ejection fraction; WBC: white blood cell; ALT: alanine aminotransferase; TG: triglyceride; TC: total cholesterol; LDL-c: low-density lipoprotein cholesterol; HDL-c: high-density lipoprotein cholesterol.

**Table 3:** Odds ratios (95% CIs) of HF according to tertiles of UA.

	Q1	Q2	Q3	*P* for trend
UA (mg/dl)		5.13-6.40		
Case/number at risk	13/73	23/71	29/72	0.011
Odds ratios (95% CI)				
Model 1	1	2.21 (1.02-4.82)	3.11 (1.45-6.67)	0.004
Model 2	1	3.01 (1.28-7.11)	4.14 (1.78-9.64)	0.001
Model 3	1	1.92 (0.70-5.24)	3.33 (1.18-9.46)	0.045
Per 1 mg/dl UA increment		1.60 (1.22-2.11)		0.001

Model 1: unadjusted OR; model 2: adjusted for sex and age; model 3: adjusted for sex, age, CRP, smoking, HBP, DM, and eGFR.

## Data Availability

The data used to support the findings of this study are included within the article.

## Supplementary Materials

Supplementary Materials Risk factors according to univariate logistic regression analysis.
