# Supplementary material for: Association between Uric Acid and In-Hospital Heart Failure in Patients with Acute Myocardial Infarction Undergoing Percutaneous Coronary Intervention
Source: Dis Markers. 2021 Jul 8;2021:7883723. doi: 10.1155/2021/7883723 (PMC8285207; doi:10.1155/2021/7883723)
Supplement: Supplementary Materials — Risk factors according to univariate logistic regression analysis. [file 7883723.f1.docx]

**Supplementary material**

|  | OR(95%CI) | P value |
| --- | --- | --- |
| Age | 1.067(1.038-1.097) | 0.000 |
| Sex(male) | 0.369(0.188-0.725) | 0.004 |
| Hypertension | 2.452(1.266-4.750) | 0.008 |
| Diabetes mellitus | 2.530(1.380-4.639) | 0.003 |
| CRP | 1.045(1.025-1.066) | 0.000 |
| BMI | 1.019(0.922-1.125) | 0.714 |
| eGFR | 0.967(0.955-0.980) | 0.000 |
| Smoking | 0.563(0.310-1.021) | 0.059 |

Risk factors according to univariate logistic regression analysis
